# False Negative ECG Device Results May Increase the Risk of Adverse Events in Clinical Oncology Trials

**DOI:** 10.1007/s43441-022-00405-0

**Published:** 2022-04-26

**Authors:** Luc Dekie, Robert B. Kleiman

**Affiliations:** Clario, 1818 Market Street, Suite 2600, Philadelphia, PA 19103 USA

**Keywords:** Oncology trials, Core lab, ECG algorithm, False negative, QRS, QTcF

## Abstract

**Background:**

Sites participating in clinical trials may not have the expertise and infrastructure to accurately measure cardiac intervals on 12-lead ECGs and rely heavily on the automated ECG device generated results for clinical decision-making.

**Methods:**

Using a dataset of over 260,000 ECGs collected in clinical oncology studies, we investigated the mean difference and the rate of false negative results between the digital ECG machine QTc and QRS measurements compared to those obtained by a centralized ECG core lab.

**Results:**

The mean differences between the core lab and the automated algorithm QTcF and QRS measurements were + 1.8 ± 16.0 ms and − 1.0 ± 8.8 ms, respectively. Among the ECGs with a centralized QTcF value > 450 or > 470 ms, 39.5% and 47.8% respectively had a device reported QTcF value ≤ 450 ms or ≤ 470 ms. Among the ECGs with a centrally measured QTcF > 500 ms, 55.8% had a device reported value ≤ 500 ms. Automated QTcF measurements failed to detect a QTcF increase > 60 ms for 53.9% of the ECGs identified by the core lab. Automated measurements also failed to detect QRS prolongation, though to a lesser extent than failures to detect QTc prolongation. Among the ECGs with a centrally measured QRS > 110 or 120 ms, 7.9% and 7.3% respectively had a device reported QRS value ≤ 110 ms or ≤ 120 ms.

**Conclusion:**

Relying on automated measurements from ECG devices for patient inclusion and treatment (dis)continuation decisions poses a potential risk to patients participating in oncology studies.

## Introduction

Accurate measurements of the ECG intervals during clinical trials are crucial for making appropriate inclusion/exclusion and dosing decisions for individual patients, as well as to allow an accurate evaluation of a new drug’s effects on heart rate, cardiac depolarization, and repolarization. The QT interval represents a global measure of the duration and uniformity of ventricular repolarization [1]. QTc thresholds (i.e. QT interval corrected for heart rate) are commonly used in clinical trial inclusion/exclusion criteria to avoid administering a drug whose QT effect has not been adequately defined to a study participant whose QTc is already prolonged [2]. The Common Terminology Criteria for Adverse Events (CTCAE) terminology, which classifies QTc prolongation criteria into 4 grades, are frequently used during clinical oncology trials to inform dosing decisions, and in particular order to avoid dosing patients whose QTc has increased since entry into the trial [3]. The QTc interval is used as a surrogate marker for the detection of increased risk of drug-induced Torsades de Pointes (TdP), a potential lethal form of polymorphic ventricular tachycardia [4, 5]. Correct measurement of the QRS duration is equally important, as drugs may delay cardiac depolarization, and study protocols may exclude patients with baseline QRS prolongation. Drug induced prolongation of the QRS interval, which represents slowing of cardiac depolarization and intracardiac conduction, may represent drug-induced block of rapid or late sodium current or a direct slowing of myocyte to myocyte conduction, and may also be a marker for increased risk of ventricular proarrhythmia [6, 7].

Cardiac intervals are assessed by recording 12-lead ECGs via a standard resting ECG device or continuous Holter recording. Most modern ECG devices include automated algorithms which can print out device generated ECG measurements and interpretive statements. Many clinicians, both in clinical practice as well as within clinical trials, do not have expertise in ECG evaluations, and may rely on the ECG device generated measurements and interpretations for clinical decision-making. The authors have reviewed cases of sudden death during which drug-induced QTc prolongation was not identified by the ECG device automated algorithm and therefore was not recognized by the prescribing physician until the ECGs were reviewed retrospectively by an experienced electrocardiographer (unpublished personal observations).

We have previously reported on the high rate of false positive QTc measurements generated by ECG device algorithms in clinical oncology trials (i.e. device reported QTc values higher than measurements performed at a core lab) [8]. The current research addresses the issues of ECG device algorithm false negative QTcF (QT interval corrected for heart rate by the Fridericia method) and QRS assessments (i.e., device reported interval measurements lower than centralized measurements), and the overall risk of relying on ECG device measurements during clinical oncology trials.

## Materials and Methods

From a set of 1,000,000 ECGs collected during a wide range of clinical drug development trials utilizing Clario (Philadelphia, PA) as a centralized core lab, we selected all ECGs collected during clinical oncology trials. All ECGs were collected digitally on calibrated ECG devices provided to the sites by Clario. ECG devices were tested using a calibrated simulator prior to shipment to the investigative sites. The ECG devices were manufactured and validated by Mortara Instruments, which utilized the VERITAS algorithm to generate ECG device measurements, or by GE Healthcare, which utilized the 12SL algorithm. ECGs were collected by the site staff and transmitted digitally to Clario or were recorded on continuous digital 12-lead Holter monitors and were stored on digital flashcards, from which Clario extracted 12-lead ECGs that were processed using the Mortara VERITAS algorithm prior to measurement by Clario personnel. The ECG device algorithm measurements were stored in the Clario database but were not available to the Clario personnel performing the ECG measurements, except for protocols that used a global median beat measurement methodology. When the latter methodology was used, the device-based measurements were available to the staff.

ECG measurements were performed in the Clario EXPERT system using either a semi-automated measurement of 3 consecutive beats on a single lead (typically lead II), or with a global median beat methodology, in which measurements are performed on a superimposition of one median beat from each lead. Nearly all ECG device algorithms, including the GE 12SL and Mortara VERITAS algorithms, use a global median beat methodology, with proprietary method of median beat formation and weighting of the various leads. With either measurement methodology, approximately 60% of ECGs required manual adjustment by Clario technicians of 1 or more caliper positions.

Measurements were performed using a semi-automated process combining an algorithm for initial caliper placement followed by review of all ECGs by a team of highly trained technician and a limited number of Clario cardiologists. The Clario technicians adjusted ECG algorithm caliper placements judged to be incorrect. As an additional step to insure correct ECG measurements, all ECGs with measurements outside the normal range, all ECGs with less than good quality, and 5% of all other ECGs selected at random went through an additional review (and if necessary, adjudication) by a second set of trained technicians. Finally, all ECGs were reviewed by a limited group of Clario cardiologists, who also had the opportunity to revise measurements.

The patient randomization status (pre- or post-randomization) was available for most ECGs, but the details of the trial design and the randomization codes were not known to Clario, and for purposes of patient confidentiality, none of the clinical characteristics nor demographics of the patients were known. Thus, the prior cardiac history and information about concomitant medications were not available.

For change from baseline QTcF (ΔQTcF) analysis, screening or baseline ECGs recorded within a 15-min time interval were considered part of the same timepoint and the average QTcF across replicates was considered as the timepoint estimate. The baseline for a given patient was defined as the pre-dose timepoint closest to dosing. Individual ECGs collected while on treatment were compared to this baseline.

## Results

A total of 261,572 ECGs had QTcF and QRS measurements from both the ECG device and the core lab available for comparison. The dataset included ECGs collected from 17,475 individual patients participating in 285 clinical oncology trials; 20,786 ECGs were recorded at screening, 19,384 ECGs were recorded at baseline, 364 ECGs at the time of randomization, 207,065 ECGs were recorded during treatment, 1694 ECGs were recorded at trial termination or during follow-up, and for 12,281 ECGs the randomization status was unknown. The age, gender, prior medical history, oncologic indication, and concurrent medications were not known for any patient. There were 217,908 ECGs measured using 3 beats in a single lead, and 43,664 measured using a global median beat methodology. The dataset for the change from baseline analysis contained 13,465 baseline timepoints and 152,066 ECGs recorded during treatment with a change from baseline QTcF value, both centrally read and based on the device algorithm.

The mean differences between the core lab and the automated algorithm QTcF and QRS measurements were + 1.8 ± 16.0 ms and − 1.0 ± 8.8 ms, respectively; on average, centralized QTcF measurements were longer than those from the ECG device algorithm, while the centralized QRS measurements were shorter (Table 1). Treatment did not seem to have a significant impact as the results pre- and post-randomization showed similar results. Across the pre-dose ECGs, the mean differences between the centrally read QTcF and QRS measurements and the automated algorithm were + 1.3 ± 14.7 ms and − 1.3 ± 8.8 ms. Across the post-dose ECGs, the mean differences between the centrally read QTcF and QRS measurements and the automated algorithm were + 1.9 ± 16.0 ms and − 0.9 ± 8.8 ms. When using the global median beat methodology, the mean differences between the QTcF and QRS measurements from the core lab and the automated algorithm were + 1.9 ± 12.6 ms and − 0.6 ± 4.8 ms, respectively, versus + 1.7 ± 16.6 ms and − 1.1 ± 9.4 ms when measurements were performed using the single lead methodology.

**Table 1:** Mean QTcF and QRS Values Across Methodologies and Randomization Status

	Number of ECGs	QTcF: automated (mean ± SD, ms)	QTcF: core lab (mean ± SD, ms)	Difference between core lab and automated QTcF values (mean ± SD, ms)	QRS: automated (mean ± SD, ms)	QRS: core lab (mean ± SD, ms)	Difference between core lab and automated QRS values (mean ± SD, ms)
3 beats, Single Lead	217,908	413.7 ± 23.8	415.5 ± 22.1	1.7 ± 16.6	93.9 ± 13.9	92.8 ± 11.3	− 1.1 ± 9.4
Global Median Beat	43,664	415.7 ± 24.9	417.5 ± 23.8	1.9 ± 12.6	95.4 ± 15.2	94.9 ± 13.9	− 0.6 ± 4.8
Pre-dose	40,532	409.9 ± 22.9	411.2 ± 21.5	1.3 ± 14.7	94.4 ± 14.4	93.1 ± 12.0	− 1.3 ± 8.8
Post-dose	208,759	414.7 ± 23.9	416.6 ± 22.3	1.9 ± 16.0	94.0 ± 14.0	93.1 ± 11.7	− 0.9 ± 8.8
All ECGs	261,572	414.1 ± 24.0	415.9 ± 22.4	1.8 ± 16.0	94.1 ± 14.0	93.1 ± 11.8	− 1.0 ± 8.8

There were many false negative ECG machine algorithm measurements. Among the 17,239 ECGs with a centralized QTcF value > 450 ms, 6817 ECGs (39.5%) had a device reported QTcF value ≤ 450 ms, and of the 3916 ECGs with a centralized QTcF > 470 ms, 1872 ECGs (47.8%) had a device reported QTcF value ≤ 470 ms (Table 2). Out of the 330 ECGs with a centrally measured QTcF > 500 ms, 184 ECGs (55.8%) had a device reported value ≤ 500 ms. Across the ECGs with a false negative QTcF machine reading, the mean centralized versus (vs.) automated QTcF values for QTcF > 450, 470 and 500 ms were 460.2 vs. 432.3 ms, 480.4 vs. 443.8 ms, and 514.6 vs 452.4 ms respectively. The standard deviation of the machine measurements was significantly higher than for the centralized measurements indicating a larger variability in the QTcF measurements.

**Table 2:** False Negatives Based on Common Exclusion and Withdrawal Criteria

Core lab measured interval (ms)	Number of central read ECGs	Number of false negatives ECGs (automated measurement below threshold)	False negative ECGs Mean ± SD value Core Lab measurement (ms)	False negative ECGs Mean ± SD value Automated measurement (ms)
QTcF > 450	17,239	6817 (39.5%)	460.2 ± 10.9	432.3 ± 23.7
QTcF > 470	3916	1872 (47.8%)	480.4 ± 11.2	443.8 ± 29.7
QTcF > 500	330	184 (55.8%)	514.6 ± 15.7	452.4 ± 47.2
QRS > 110	12,177	964 (7.9%)	116.2 ± 7.3	104.8 ± 8.2
QRS > 120	8543	624 (7.3%)	126.8 ± 7.4	113.2 ± 10.6

The differences between the centralized and ECG machine QRS measurements were of smaller magnitude. Out of the 12,177 ECGs with a centrally measured QRS > 110 ms, 964 (7.9%) had a device reported QRS value ≤ 110 ms, and of the 8543 ECGs with a centrally measured QRS > 120 ms, 624 ECGs (7.3%) had a device reported QRS ≤ 120 ms (Table 2). Across the ECGs with a false negative QRS machine measurement, for centralized QRS measurements of > 110 and > 120 ms, the mean centralized and machine measurements were 116.2 vs. 104.8 and 126.8 vs. 113.2 ms respectively. Similar trends were seen when evaluating only ECGs recorded prior to the first drug administration (Table 3).

**Table 3:** False Negatives Based on Common Exclusion Criteria, Pre-dose ECGs Only

Core lab measured interval (ms)	Number of central read ECGs	Number of false negatives ECGs (automated measurement below threshold)	False negative ECGs Mean ± SD value Core Lab measurement (ms)	False negative ECGs Mean ± SD value Automated measurement (ms)
QTcF > 450	1661	701 (42.2%)	460.1 ± 10.3	433.0 ± 23.5
QTcF > 470	345	189 (54.8%)	480.9 ± 11.1	445.4 ± 27.7
QTcF > 500	28	17 (60.7%)	515.2 ± 10.3	444.8 ± 58.4
QRS > 110	1891	122 (6.5%)	116.3 ± 7.5	105.7 ± 4.9
QRS > 120	1318	82 (6.2%)	126.5 ± 6.6	113.8 ± 7.1

Table 4 shows the agreement between the core lab and device algorithm measurements when grading the ECGs using the CTCAE QTc prolongation criteria. The agreement was 55.6, 38.5 and 44.2% for grade 1, 2 and 3 respectively. ECGs measured at the core lab using the global median beat showed a greater agreement with the device readings compared to the ECGs measured using the 3 beats on a single lead methodology. For the global median beat methodology, the agreement was 78.0, 60.1 and 51.2% for grade 1, 2 and 3 respectively, versus 49.4, 29.7 and 39.9% for the three beats on a single lead methodology.

**Table 4:** Agreement Between the Detection of CTCAE Defined QTcF Prolongation by Centralized and Device Measurements

Core lab measurement methodology	Total # ECGs	Core lab			ECG device		
		Grade 1 (450–480 ms)	Grade 2 (481–500 ms)	Grade 3 (> 500 ms)	Grade 1 (450–480 ms)	Grade 2 (481–500 ms)	Grade 3 (> 500 ms)
Global Median Beat	43,664	3590	424	127	2802 (78.0%)	255 (60.1%)	65 (51.2%)
3 beats, Single Lead	217,908	13,102	1037	203	6476 (49.4%)	308 (29.7%)	81 (39.9%)
Total	261,572	16,692	1461	330	9278 (55.6%)	563 (38.5%)	146 (44.2%)

Based on the centralized measurements, the QTcF change from baseline (ΔQTcF) was > 30 ms for 10,857 ECGs (Table 5). The device algorithm based ΔQTcF based was ≤ 30 ms for 48.3% of these cases. Out of 707 ECGs for which the core lab identified a ΔQTcF > 60 ms, 381 ECGs (53.9%) had ΔQTcF ≤ 60 ms based on the device algorithm measurements.

**Table 5:** Comparison of Centralized and Device Measurements-QTcF Change from Baseline

Centralized measurement	Number of ECGs	Device measurement < threshold
ΔQTcF > 30 ms	10,857	5241 (48.3%)
ΔQTcF > 60 ms	707	381 (53.9%)

**Fig. 1:**
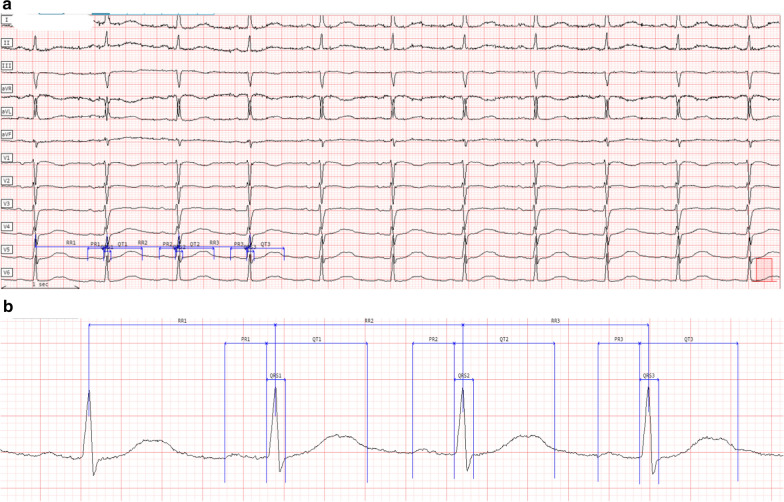
ECG with QT and QTc Undermeasured by the ECG Device; Centralized Measurements on a Single Lead. **a** 12-lead ECG; **b** magnification of ECG core lab caliper placements (measured on lead V5). Centralized measurement: QTcF 506 ms (mean QT 492 ms); Device algorithm QTcF 354 ms (QT 344 ms)

**Fig. 2:**
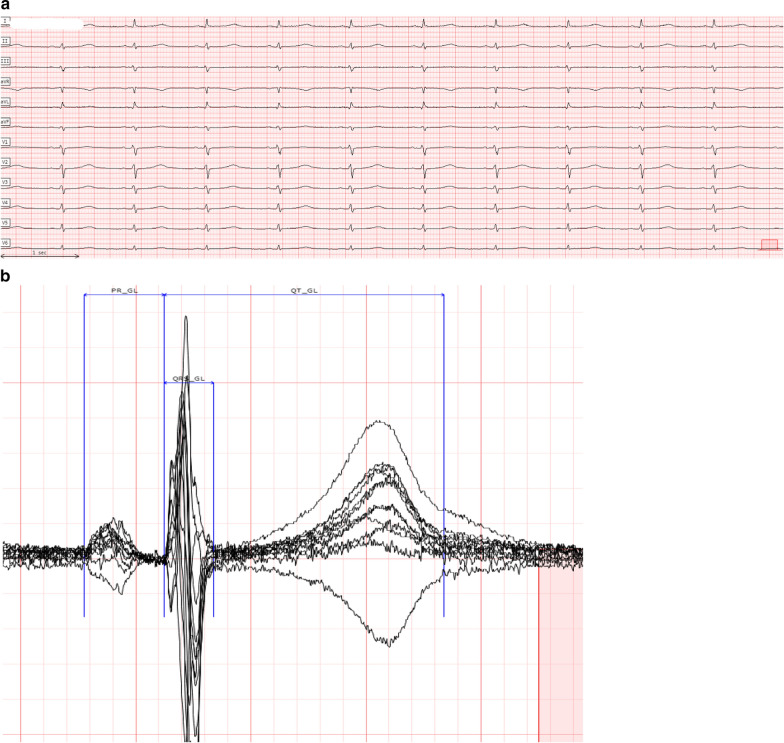
ECG with QT and QTc Undermeasured by ECG Device; Centralized Measurements Performed on a Global Median Beat. **a** 12-lead ECG; **b** magnification of ECG core lab caliper placements on global median beat. Centralized measurement: QTcF 500 ms (mean QT 487 ms); Device algorithm QTcF 403 ms (QT 392 ms)

**Fig. 3:**
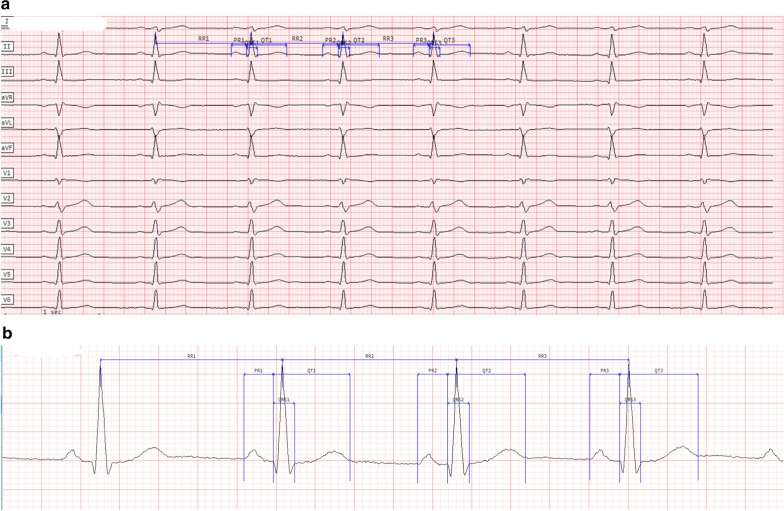
ECG with QRS Duration Underreported by the ECG Device; Centralized Measurements on a Single Lead. **a** 12-lead ECG. **b** Magnification of ECG core lab caliper placements (measurements on lead II). Centralized measurement: mean QRS 101 ms; Device algorithm measured QRS 39 ms

Figures 1 and 2 show examples of ECGs where the end of the T-wave was incorrectly identified by the ECG machine algorithm, resulting in under-reporting of the QT and QTc intervals. Figure 1 shows an ECG where the QT was centrally read on three consecutive beats on a single lead. The mean centralized QT measurement was 492 ms, while the device algorithm undermeasured the QT at 344 ms. Figure 2 shows an ECG for which the centralized measurements were performed using the global median beat methodology. The ECG machine algorithm under-measured the QT interval by 95 ms. Figure 3 shows an ECG for which the ECG machine algorithm markedly undermeasured the QRS duration. The mean centrally measured QRS duration was 101 ms, while the ECG machine algorithm reported a QRS duration of 39 ms.

## Discussion

The accurate evaluation of the cardiovascular safety profile is an important step during the development of any new investigational drug. Depending on the direct and indirect mode of action of the drug, tests may include cardiac imaging, analysis of cardiac biomarkers, blood pressure assessments and electrocardiographic monitoring [9, 10]. Although there are numerous different classes of cardiac toxicities, among the most serious is ventricular proarrhythmia which may result in sudden cardiac death. The mechanism for this rare but lethal event is now known to be Torsades de Pointes (TdP), an unusual type of ventricular tachycardia that has a characteristic morphology and is a consequence of alterations in ventricular repolarization. For non-antiarrhythmic drugs that produce TdP, the incidence is low enough that it is not feasible to detect an excess of TdP during clinical trials, and drug developers have therefore been forced to rely on surrogate markers for assessing a new drug's risk of producing TdP. The surrogate marker that is currently utilized for the detection of increased risk of drug-induced TdP is prolongation of the QTc interval [4, 5]. All drugs that are known to produce TdP have been demonstrated to prolong QTc (as measured on the surface ECG) despite different chemical structures and potentially different mechanisms of prolonging cardiac repolarization. The current paradigm for detection of drug-induced QTc prolongation, as described in the International Conference for Harmonization (ICH) E14 Guidance for Industry and its subsequent Q&A releases, relies on detection of a drug-induced mean QTc prolongation of about 5 ms (as evidenced by an upper bound of the 95% confidence interval around the mean effect on QTc of 10 ms) [11]. For drugs with relatively wide therapeutic indices, the QTc assessment may be performed in a dedicated QT/QTc study or during the single and multiple ascending dose studies performed early in a drug's development. These studies are usually performed in healthy volunteers under highly controlled clinical trial settings and involve serial replicate ECGs collection time-matched to the PK sampling timepoints, the use of precise measurement techniques and limitations in the use of concomitant medications [12]. Many oncologic drugs, however, cannot be administered to healthy volunteers in supratherapeutic or even therapeutic doses, and QTc assessments must therefore be performed in the clinical trials performed with oncology patients who are typically older, may have additional comorbidities, are prone to electrolyte shifts, and on average, have a higher QTc than healthy volunteers [13]. The precision of QTc measurements is therefore very important as greater precision results in increased statistical power of the trial to accurately assess a drug's effect on QTc.

Twelve-lead ECGs are collected in nearly all clinical trials during the screening or baseline assessments and during the treatment phase. The inclusion/exclusion criteria typically include QTc (and to a lesser extent QRS) threshold criteria to avoid administering a drug whose effect on cardiac conduction has not been adequately defined to a subject with preexisting QTc or QRS prolongation. Many oncologic agents are known to prolong the QTc interval. The QRS is an integral part of the QT interval, and therefore drug-induced QRS prolongation can result in a direct increase in the QT interval, related to the increased QRS duration, independent of any real effect on ventricular repolarization [14]. The CTCAE classification guideline describes QTc prolongation criteria as grade 1 (QTc 450–480 ms), grade 2 (QTc 481–500 ms), grade 3 (QTc > 500 ms or QTc change from baseline > 60 ms) and grade 4 (Torsade de pointes; polymorphic ventricular tachycardia; signs/symptoms of serious arrhythmia) [3]. In many oncology studies, a grade 2 QTc prolongation may result in withholding drug dosing until the QTc prolongation resolves. A false positive report of QTc prolongation based on the ECG machine algorithm values may lead to increased ECG monitoring or withholding of doses. In a clinical oncology trial, unnecessary withholding of doses may have deleterious effects both for the patient as well as for the drug development program. During the dose escalation portion of a Phase I oncology trial, this may also affect decisions about dose escalation. Grade 3 QT prolongation may lead to patient discontinuation from the trial or may be considered a dose limiting toxicity that precludes further dose escalation. The risk of TdP is not a linear function of the duration of the QTc interval, nor of the extent of QTc prolongation during drug therapy [15, 16]. However, progressive prolongation of the QTc interval increases the risk for TdP, and the risk increases markedly when the QTc interval exceeds 500 ms [17–19]. Data from Congenital Long QT Syndrome studies indicate that a QTc > 500 ms is associated with a much higher risk for TdP. Likewise, case reports and small series of patients with drug-induced TdP show similar increased risk when the threshold of QTc > 500 ms is exceeded [20].

False negative results—underreporting of QTc prolongation by automated ECG algorithms—poses even greater risks. A false negative QTc value (a QTc value reported by the ECG machine algorithm that is substantially lower than the true QTc value) exposes the patient to the risk that further drug-induced QT prolongation may result in TdP, potentially with lethal consequences. While false positive findings tend to be disruptive for the site and patient, they rarely impact patient safety, and their effects can be mitigated following recognition that the finding is a false positive. In contrast, false negative results may expose patients to safety risks and may result in patient deaths before they are recognized.

Accurate cardiac intervals assessments are thus of critical importance in oncology trials, both to maintain patient safety as well as to allow program wide assessments of the QTc effects of a new drug. Many clinical trials in oncology do not utilize a central ECG lab to perform the cardiac interval measurements and rely upon the sites to evaluate the ECGs. Many clinicians, both in clinical practice as well as within clinical trials, do not have expertise in ECG evaluations [21, 22]. Furthermore, those who are familiar with measuring ECG intervals may still use the ECG machine algorithm measurements, which can contain errors and cannot match the precision of core lab measurements performed using digital ECG waveforms measured at high magnification [23, 24]. Most ECGs are printed with a paper speed of 25 mm/s and have a pen width of 5–10 ms. Using metal calipers, the resolution of QT measurements ranges from 10 to 20 ms. In contrast, centralized interval measurements of digital ECG waveforms can typically achieve a resolution of 1 ms.

We previously reported on the presence of false positives when comparing ECG device measurements with device measurements. The present study extends these results by evaluating ECG device algorithm false negative QTc and QRS assessments, by analyzing a dataset of 261,572 ECGs collected during oncology clinical trials.

The mean QTcF and QRS differences between the core lab and the device algorithm measurements were small, + 1.8 and − 1.0 ms respectively. On average the core lab QTcF measurements were slightly longer than those from the ECG device algorithm, while the core lab’s QRS measurements were shorter, independent of the measurement methodology used by the core lab or randomization status. However, the confidence bounds around these point estimates were larger based on device algorithms, reflecting the large numbers of automated measurements that were significantly too high or too low. Exclusion thresholds commonly used in clinical trials include QTcF > 450 ms and QTcF > 470 ms. Our analyses found that ~ 40% and ~ 48% of the ECGs with a centrally measured QTcF value > 450 and > 470 ms, respectively, had a device measurement below these thresholds. Furthermore, the differences in measurements often were not trivial. Among ECGs with a centrally measured QTcF > 450 ms, the mean centrally measured QTcF was 460 ms, compared to a mean automated measurement of 432 ms. For the ECGs with centrally measured QTcF > 470 ms, the mean centrally measured QTcF was 480 ms compared to a mean automated measurement of 444 ms. These effects were independent of the investigational drug being evaluated, as the results were similar for both pre-randomization and post-randomization ECGs. For the ECGs with a centralized QTcF > 500 ms, 56% had an automated measurement ≤ 500 ms, i.e., the CTCAE grade 3 QTc prolongation agreement was 44%. The mean QTcF for these ECGs was 515 and 452 ms for centralized and automated measured QTcF, respectively, with a standard deviation of 16 and 47 ms, respectively.

ECG machine automated measurements also failed to detect many instances in which QTcF had increased substantially compared to baseline. Out of 707 ECGs for which the core lab identified a QTcF increase from baseline > 60 ms, only 46.1% were correctly identified by the ECG machine measurements. Reliance on ECG machine measurements may therefore result in patients who have already had a large QTc increase continuing to receive a drug that may have been responsible for the QTc prolongation. The large categorical and central tendency difference between the centrally read and automated measurements may be due to these ECGs being more noisy or having challenging T waves (flat or biphasic T wave), which confound the ability of the algorithms to accurately determine the end of the T wave. However, as illustrated in Figs. 1, 2 and 3, ECG algorithms may generate false negative QRS and QT measurements even for good quality ECGs.

False negative QTc data may result in the inclusion in a trial of patients who may be at increased risk when receiving an investigational drug whose effect on repolarization is not yet known. Under-measurement of the QRS duration may also expose patients to proarrhythmic risk for both QTc and QRS prolonging drugs. Since ECG devices algorithms use the QRS duration as a criterion to determine the presence of a bundle branch block, complete bundle branch block may be missed by sites who heavily rely on their ECG device generated measurements and interpretations for clinical decision-making.

### Limitations

There are several limitations to this study. This investigation was performed retrospectively, and only included ECGs from trials that utilized central core lab measurement of ECGs. It is possible that these trials utilized central measurement of the ECGs because prior trials of the investigational agent had already demonstrated a higher than expected rate of failures of the ECG machine algorithmic measurements—in other words, referral bias.

However, there appeared to be little difference between our findings in pre- and post-randomization ECGs, suggesting that the investigational agents tested in these trials did not contribute to the differences between the device and central core lab ECG measurements. We also evaluated all consecutive ECGs collected over a long time interval, including many different trials with differing designs, patient populations, and therapies with the aim of avoiding any selection bias. Nevertheless, it remains possible that the trials involved in this study were chosen for centralized ECG processing because they recruited patients with more complex cardiac disease and ECGs and, thus, might not be representative of the average oncology patient. In addition, since we were blinded to the patient demographics, we were unable to stratify the findings based on factors that may affect ECG findings, such as age, gender, or prior history.

We also evaluated measurements from ECG devices from only 2 manufacturers, though these utilize the 2 ECG measurement algorithms most commonly used in the ECG devices used in clinical trials and our results, not published, do not indicate a difference between the algorithms in the frequency of false negative QTc findings. Several studies comparing the different ECG machine algorithm performance have shown relatively small differences between the accuracy of these algorithms and the other commercially available algorithms [25].

We believe that the central core lab measurements were more accurate than the ECG device measurements since all centralized measurements were confirmed by at least 2 and often 3 different individuals who were blinded to the patient randomization and to the design and conduct of the clinical protocols.

## Conclusion

Our analysis shows that the use of automated measurements from ECG devices poses a potential risk to patients participating in oncology studies. False negative QTcF and QRS automated measurements may lead to the inappropriate inclusion of high-risk patients into a trial and during a trial may lead to patient exposure to a drug that has already produced QTc or QRS prolongation. ECGs should therefore always be carefully reviewed and measured at the site or at a central core lab to detect measurement errors.
